# An Easy and Single-Step Biosynthesis of WO_3_ with High Photocatalytic Degradation Activity for Dye Degradation

**DOI:** 10.3390/nano15131036

**Published:** 2025-07-03

**Authors:** Azza A. Al-Ghamdi, Reema H. Aldahiri, Elham A. Alzahrani, Naha Meslet Alsebaii, Sumbul Hafeez, Shafiul Haque, Poonam Dwivedi, Seungdae Oh

**Affiliations:** 1 Department of Chemistry, College of Science, University of Jeddah, Jeddah 21959, Saudi Arabia; aaalgamdi@uj.edu.sa (A.A.A.-G.); rhal-dhahery@uj.edu.sa (R.H.A.); 2 Department of Chemistry, College of Science, University of Ha’il, Ha’il 81451, Saudi Arabia; elh.alzahrani@uoh.edu.sa; 3 Department of Chemistry, Faculty of Science, King Abdulaziz University, P.O. Box 80200, Jeddah 21589, Saudi Arabia; nalsebaii@kau.edu.sa; 4 Department of Civil and Environmental Engineering, Villanova University, Villanova, PA 19085, USA; sumbulhafeez03@gmail.com; 5 Department of Materials Science and Engineering, Indian Institute of Technology Delhi, Hauz Khas, New Delhi 110016, India; 6 Department of Nursing, College of Nursing & Health Sciences, Jazan University, Jazan 82911, Saudi Arabia; shafiul.haque@hotmail.com; 7 School of Medicine, Universidad Espiritu Santo, Samborondon 091952, Ecuador; 8 Department of Chemistry, Ramjas College, University of Delhi, Delhi 110007, India; 9 Department of Civil Engineering, College of Engineering, Kyung Hee University, Yongin 17104, Republic of Korea

**Keywords:** water, dye, methylene blue, photodegradation, photocatalyst, WO_3_

## Abstract

In the present study, a photodegradation technique was employed for the removal of methylene blue dye from aqueous solution using a tungsten oxide-based photocatalyst. The photocatalyst was synthesized via a green synthesis route utilizing a plant extract (PE) under acidic conditions. The synthesized photocatalyst was characterized by various spectroscopic and microscopic techniques that confirmed the presence of various functional groups on the catalyst surface and revealed a narrow bandgap of ~3.0 eV. The synthesized particles exhibited a nanoscale dimension ranging from 10 to 15 nm. The photocatalytic activity of the material was evaluated under ultraviolet light, visible light, and sunlight irradiation, demonstrating the efficient degradation of methylene blue under all light sources. Furthermore, catalysis reusability studies indicated excellent stability, with consistent photocatalytic performance observed after five successive cycles.

## 1. Introduction

The presence of synthetic dyes in water has emerged as a critical environmental concern, primarily due to the increasing discharge of industrial effluents as industrial activities expand [1,2]. Dyes are extensively utilized across various industries for coloring products, and these compounds often enter aquatic bodies through untreated or inadequately treated wastewater. Once incorporated into water bodies, dyes hinder sunlight penetration, adversely affecting water quality and disrupting aquatic ecosystems (flora and fauna). Moreover, dye-contaminated water causes serious health risks to humans who rely on these water sources [3,4,5,6]. The toxicity of dyes is largely attributed to their complex molecular structure [6,7], which includes benzidine and is known for being carcinogenic and mutagenic [7]. Among various synthetic dyes, methylene blue (MB) is one of the most used and studied [8]. MB is highly toxic and has been associated with carcinogenic effects and other adverse health effects [8,9]. Therefore, it is imperative to either prevent the release of such a dye into the environment or develop effective strategies for its removal from contaminated water sources [10]. Numerous techniques have been adopted for the removal of MB from aqueous systems, including adsorption [11], photocatalyst degradation [12], oxidation [13], coagulation [14], filtration [15], and membrane filtration [16]. Among these, photocatalytic degradation has gained significant attention due to its ability to not only remove the dye but also degrade it into less harmful byproducts under light radiation (solar, visible light, or UV light) [17,18,19]. The materials used in this process are referred to as photocatalysts [17,18,19], and their optical performance is closely related to their energy band gap [20]. Materials with wide band gaps exhibit limited photocatalytic activity under visible light or sunlight, whereas those with narrow band gaps can effectively harness these light sources for degradation reactions [21]. A wide range of photocatalytic materials have been used, such as metal oxide-based photocatalysts, which demonstrate themselves to be a promising photocatalyst candidate due to their stability, availability, and efficient photocatalytic properties [22]. 

In previous studies [23,24], TiO_2_-based photocatalysts were under ultraviolet (UV) light, which increased the overall cost of the degradation of dye. Therefore, there is an urgent necessity for the synthesis of low-cost photocatalysts. To overcome these limitations, nanomaterials (NMs) receive immense attention due to their significant optical properties and are referred to as nanophotocatalysts (NPCs) [25,26,27]. The efficiency of NPCs is significantly influenced by their band gap energies and the semiconductor’s electronic energy level. Furthermore, copper oxide (CuO) nanoparticles (NPs) have garnered attention as promising NPC candidates due to their high thermal stability, excellent conductivity, cost-effectiveness, superconducting behavior at elevated temperatures, and low toxicity [28,29]. Nevertheless, the practical applications of pristine CuO are hindered by a high electron–hole recombination rate, which substantially limits its photocatalytic efficiency [30]. To address this limitation, various strategies have been employed, notably the construction of heterojunctions [12,31], such as CdS/ZnO [32], CdS/SnO_2_ [33], α-Fe_2_O_3_/BaTiO_3_ [34], CuNiFe_2_O_4_/g-C_3_N_4_ [35], and hybrid g-C_3_N_4_/ZnO-W/Cox [36], which have been demonstrated to be useful for MB dye removal. However, these systems often involve expensive precursors and complex fabrication techniques, which hinder large-scale implementation. Consequently, there is a critical need to explore cost-effective and easily synthesizable alternative photocatalytic materials for the efficient photodegradation of MB dye.

Tungsten oxide (WO_3_) nanostructures (NSs) have received considerable interest for a wide range of applications, including sensors, photochromic applications, electrochromic applications, and notably photocatalysis, due to their inherent electrical conductivity [37,38,39,40]. WO_3_ is an n-type semiconductor with a tunable band gap reported to be in the range of approximately ~2.6 to ~3.2 eV [41]. 

Conventionally, WO_3_ has been synthesized through various chemical and physical techniques [42,43,44,45,46]. However, in comparison to conventional chemical approaches, biosynthetic material utilizing plant extracts (PEs) has emerged as a more sustainable and cost-effective alternative [47,48]. The biosynthetic approach offers several advantages, including the production of photocatalysts with a high surface area, abundant surface functional groups, and tunable chemical, physical, optical, and electronic characteristics [47,48]. Despite these advantages, the use of a PE for the biosynthesis of WO_3_ remains relatively unexplored, with only limited studies reported to date [47,48,49]. Considering the extensive potential of WO_3_ in photodegradation applications, there is a compelling need to investigate and optimize the selection of appropriate plant sources to enhance the efficiency and reproducibility of the biosynthesis process. 

In this study, WO_3_ nanostructures were synthesized via a green synthetic approach utilizing *Kigelia pinnata* (Sausage tree) leaves extract. *Kigelia pinnata* is a species native to sub-Saharan Africa [50,51]. Though plant-mediated synthesis has been explored for various metal oxides, the use of *Kigelia pinnata* leaf extract for the synthesis of perovskite nanoparticles remains largely unexplored, with only limited reports available [51]. This renders the current investigation both novel and significant. 

The biosynthesized WO_3_ photocatalyst was systematically evaluated for its efficacy in the degradation of MB dye under three different irradiation conditions, such as UV light, visible light, and sunlight. A relative demonstration of photocatalytic activity under these irradiation sources was performed, representing, to the best of our knowledge, the first study of its kind using *Kigelia pinnata*-derived WO_3_. Furthermore, radical scavenging experiments were conducted to elucidate the active species involved in the photodegradation process. The thermal regeneration capability of the photocatalyst was also assessed to determine its reusability and stability over multiple cycles.

## 2. Experiments

### 2.1. Material Required

Sodium tungsten dihydrate (Purity = 99%), concentrated hydrochloric acid (Purity = 37%), and methylene blue (vendor-mentioned purity of 99.0%) were procured from Sigma Aldrich, St. Louis, MO, USA. *Kigelia pinnata* leaves were collected from the local area, Jeddah, Saudi Arabia.

### 2.2. Preparation of Kigelia Pinnata Leaf Extract

A total of 250 g of *Kigelia pinnata* leaves was collected and washed several times with deionized water. The washed leaves were then dried for 2 weeks in sunlight. Thereafter, using a mixer grinder, the dried leaves were ground into a fine powder. Then, ~2.5 g of the prepared leave powder was mixed in 100 mL of warmed deionized water (which was preheated to 70 °C). The temperature of the aqueous mixture was then raised to 90 °C on a magnetic stirrer and continuously stirred for 25 min at 90 °C to obtain a concentrated extract of the leaves. The extract obtained was filtered using Whatman paper no. 1 and stored in a refrigerator for further analysis.

### 2.3. Biosynthesis of WO_3_ NPs

Firstly, 100 mL deionized water was taken in a 250 mL beaker, and then 3.2 g (0.1 M) of sodium tungstate was dissolved in it on a magnetic stirrer with a stirring rate of 120 rpm. After complete dissolution of sodium tungstate, 40 mL freshly prepared *Kigelia pinnata* P.E. was added along with 5 mL concentrated HCl under constant stirring conditions (120 rpm) at 80 °C for an hour. Afterwards, a brown precipitate formation was observed, followed by a transition in color from brown to gray after an hour, indicating the completion of the preparation process. The gray color precipitate was separated using a centrifuge machine (at 5000 rpm) and then dried at 60 °C for 24 h in a vacuum oven. The dried sample was then calcined in a muffle furnace at 400 °C for 2 h. The calcined sample was stored and used for further physicochemical characterization and photodegradation application. 

### 2.4. Preparation of Stock Solution of Dye

The MB dye solution was prepared by dissolving 1000 mg of MB in 1000 mL of deionized water at room temperature and neutral pH. The prepared stock solution of MB was of 1000 ppm (i.e., 1000 mg/L), which was further diluted as per experimental conditions using the dilution law N_1_V_1_ = N_2_V_2_. 

### 2.5. Characterization of WO_3_

Various spectroscopic and microscopic techniques were performed to characterize the prepared sample. The function group properties of the prepared photocatalyst were analyzed by Fourier transform infrared (FTIR) using a Tensor 37, Bruker, FTIR spectrometer from the 400 to 4000 cm^−1^ wavenumber range. X-ray diffraction (XRD) analysis was performed to analyze the crystal structure and phase of the prepared sample using a Rigaku Smart Lab Guidance, Rigaku, XR diffractometer.

The crystallite size of the sample was also determined using the Scherrer equation (Equation (1)) [52].

(1) D = Kλ/βCosθ

Here, *D* = crystallite size; *K* = shape factor; *λ* = X-ray wavelength (0.154 nm); *β* = full-width half maxima of the corresponding peak; and θ = diffraction angle. 

The scanning electron (SE-NOVA NANOSEM-450, FEI) and transmittance electron (TE- F30 S-Twin, Technai) microscopes were used for microscopic (SEM and TEM) analysis, which describes the morphology of the surface and inside information of the NPs. An energy dispersive X-ray spectrometer (EDS) connected to the SEM microscope and a selected area electron diffraction (SAED) spectrometer connected to the TEM microscope were used to determine the element composition and planes of the WO_3_ photocatalyst, respectively. A U3900, Hitachi, ultraviolet (UV)–visible (Vis) spectrophotometer (Hitachi, Tokyo, Japan) was used to measure the band gap of WO_3_ and for the absorption analysis of the MB solution. 

### 2.6. Photocatalytic Degradation of Dye

In this study, the WO_3_ that formed was used as a photocatalyst. As discussed above, the high content of dyes, especially that of MB dye, in water is hazardous to human health. Hence, in the present study, WO_3_ was used for the photocatalytic degradation of MB dye. The dye degradation was examined under UV light irradiation and compared with visible light and sun light irradiation for 120 min with and without the WO_3_ photocatalyst. A medium-pressure mercury (Hg) lamp (energy consumption = 450 W) was used for these photocatalytic experiments. The dye was degraded from a concentration of 20 mg/liter using an amount of 2 g/liter of WO_3_ at pH 7 and 27 °C, respectively. The degradation was first carried out with or without the WO_3_ catalyst in UV light irradiation, as well as in the dark. The dye was shaken (at 200 rpm) with the catalyst for 10 min in order to initiate the adsorption process, after which the degradation process was started.

### 2.7. Spectroscopic Analysis and Degradation Efficiency

Firstly, to determine the initial concentration of the solution (i.e., *C*_o_), the absorbance was measured at different concentrations (10–50 mg/L) of the MB solution at 600 nm λ-max (absorbance maximum of MB) using a UV–Vis spectrophotometer (U3900, Hitachi). Then, a calibration curve was plotted using these absorbance values and followed by the Beer–Lambert linearity to an extent of about 99.8%. The solution with the obtained initial concentration was subjected to a degradation experiment in the presence and absence of the WO_3_ photocatalyst as per the above-mentioned experimental conditions (Section 2.6), and then the absorbance of the MB solution after the degradation experiment (i.e., filtrate) was determined under the same spectroscopic conditions. These final absorbances were used for measuring the final concentration of the MB solution (*C_t_*) after the degradation experiments. 

The degradation efficiency (%) of MB using WO_3_ was calculated by providing the initial and final concentration in Equation (2) [53].

(2) $$\mathrm{Photodegradation}\;\mathrm{efficiency}\;(\%)\;=\;(C_{o}\;-\;C_{t}\;/C_{o})\;\times \;100$$

### 2.8. Kinetic Study

To evaluate the photocatalytic degradation behavior of organic contaminants, two kinetic models were employed: the pseudo-first-order kinetic model and the diffusion model. 

The pseudo-first-order kinetic model is widely applied to describe the photocatalytic degradation of organic pollutants, particularly when they are present at low concentrations (typically a few mg/L) in aqueous media [54]. This model states that the degradation rate under light irradiation is primarily dependent on the pollutant concentration, while the photocatalytic concentration remains constant throughout the reaction.

In contrast, the diffusion kinetic model of photodegradation describes the transport of contaminants (such as dyes) toward the photocatalyst surface in an aqueous medium, followed by their adsorption and subsequent photoreaction under light radiation. The degradation rate in this case is influenced by the diffusion of contaminants to the photocatalyst surface, as well as the efficiency of the surface photoreaction.

The photodegradation rate is defined by these models, which are represented by Equations (3) and (4), respectively [55].

(3) $$ln(C_{o}/C_{t})\;=k_{1}t$$

(4) $$C_{t}^{0.5}-C_{o}^{0.5}\;=\frac{k_{2}}{2}t$$

where *C_o_* and *C_t_* are the initial and final concentrations of the contaminant in aqueous solution, *t* is the irradiation time, and *K_1_* and *K_2_* are the pseudo-first-order and diffusion kinetic rate constants, respectively.

### 2.9. Regeneration of Photocatalyst

To evaluate the reusability and practical applicability of the photocatalyst, a regeneration process was carried out using thermal treatments. The photocatalyst was thermally heated at three temperatures, 150, 250, and 350 °C. Following thermal treatment, the photocatalyst was allowed to cool to room temperature and subsequently washed thoroughly with deionized water to remove any residual impurity. The resulting photocatalyst was then dried in a hot air oven at 90 ° for 24 h. The regenerated photocatalyst was reused in five consecutive cycles to assess its catalytic activity and stability performance over repeated use [56].

### 2.10. Free Radical Scavenging Experiments

Free radical scavenging is an important step to understand the mechanism of photodegradation of any organic molecule on the photocatalyst surface. This experiment determines the free radical neutralization activity of a compound that acts as a trapping agent (or scavenger) for free radicals like hydroxide (^•^OH) and superoxide (^•^O_2_^−^) ones. Depending on the free radical scavenging activity, this experiment reveals which radical plays a key role in photodegradation. The free radical scavenging test was conducted using 0.25 mM of tert-butyl alcohol (t-BuOH: hydroxide free radical; ^•^OH) and p-benzoquinone (BQ: superoxide free radical; ^•^O_2_^−^) [57].

## 3. Results and Discussion

### 3.1. Results of Characterization Analysis

#### 3.1.1. Crystal Phase and Size Determination

**Figure 1:**
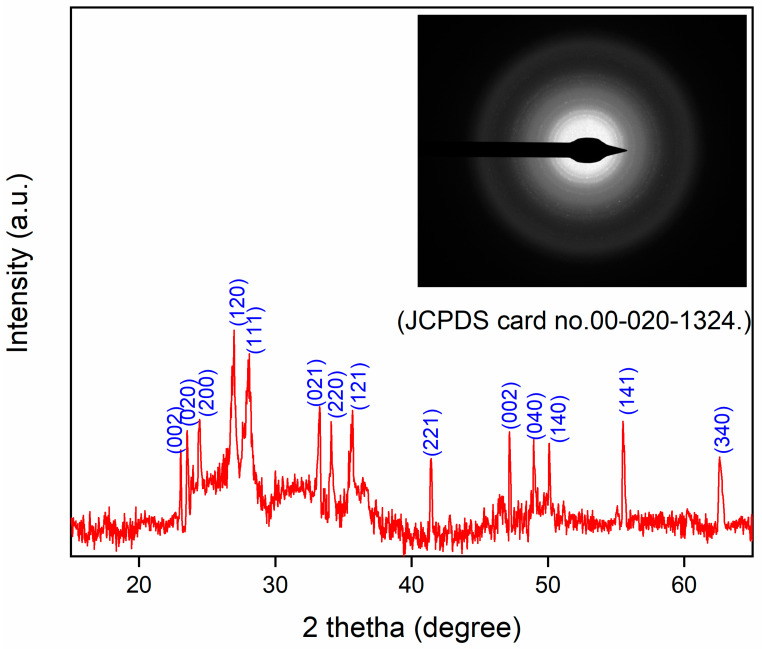
XRD pattern of prepared WO_3_ (inset: SEAD pattern of WO_3_).

XRD analysis was carried out to determine the phase and crystal structure of WO_3_. The XRD analysis was performed from 10° to 80° (Figure 1). WO_3_ mainly displays the monoclinic phase through the analyzed *2θ* values 23.14°, 23.58°, 24.44°, 26.96°, 28.08°, 33.72°, 34.08°, 35.66°, 41.44°, 47.16°, 48.94°, 50.10°, 55.46°, and 62.68°, with the corresponding planes (002), (020), (200), (120), (111), (021), (220), (121), (221), (002), (040), (140), (141), and (340) as represented in the XRD pattern. This result can be confirmed by JCPDS card No. 00-020-1324 [47] and previous studies [48,58,59]. The size of the formed WO_3_ was also measured through XRD; for this, the Scherrer equation was used (Equation (1)). The WO_3_ size came out to be ~16 nm. The major reason for the small size of the crystal was the stabilization of WO_3_ NPs through the functional group of phytochemicals of the PE.

#### 3.1.2. Morphology and Elemental Analysis

**Figure 2:**
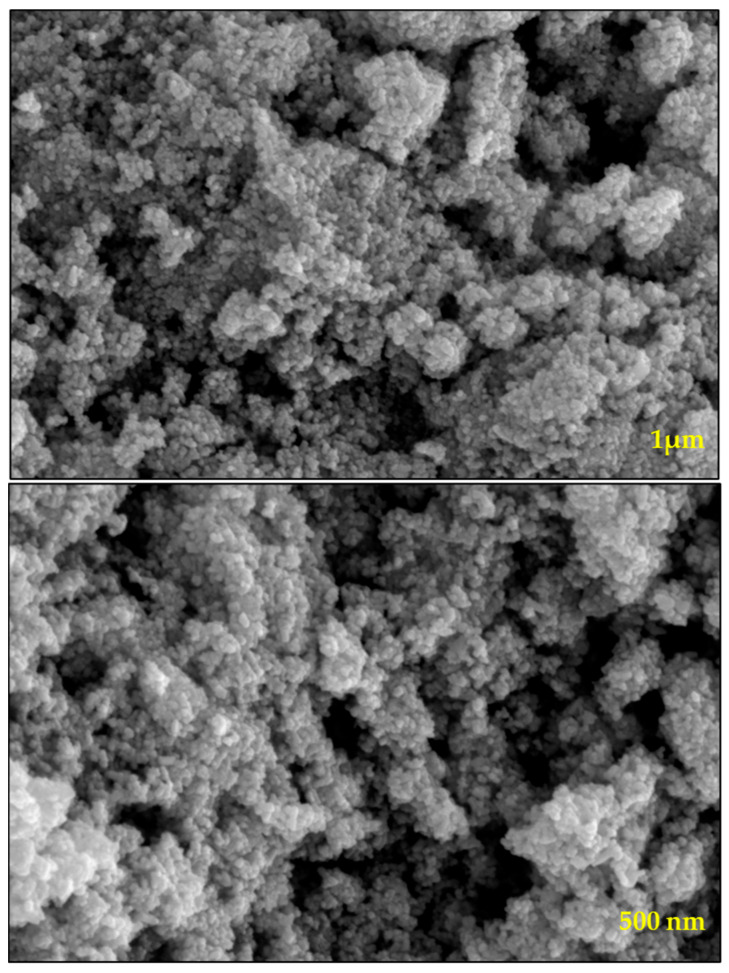
SEM images of prepared WO_3_ at different magnifications.

**Figure 3:**
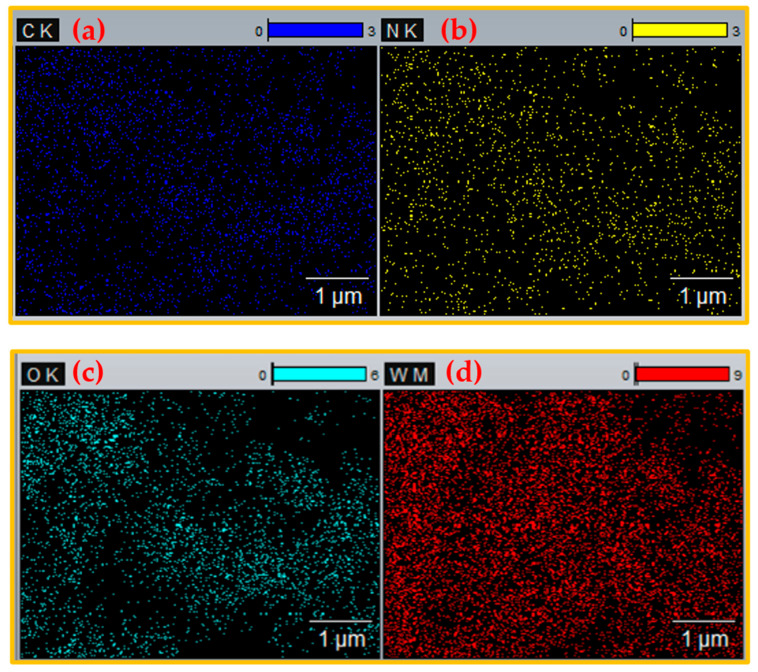
Elemental mapping of (**a**) carbon, (**b**) nitrogen, (**c**) oxygen, and (**d**) tungsten in WO_3_ NPs.

**Figure 4:**
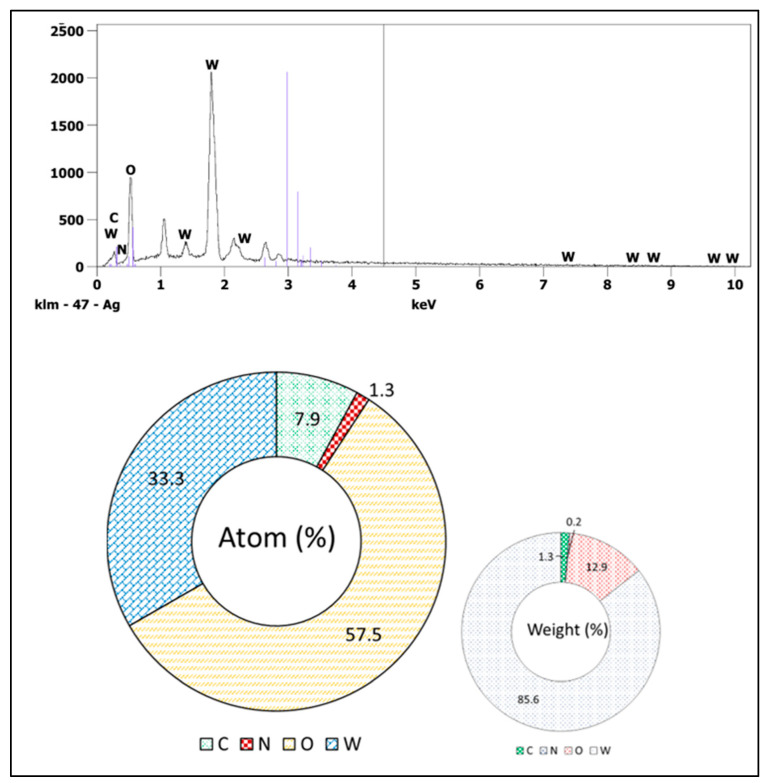
EDX analysis of WO_3_.

**Figure 5:**
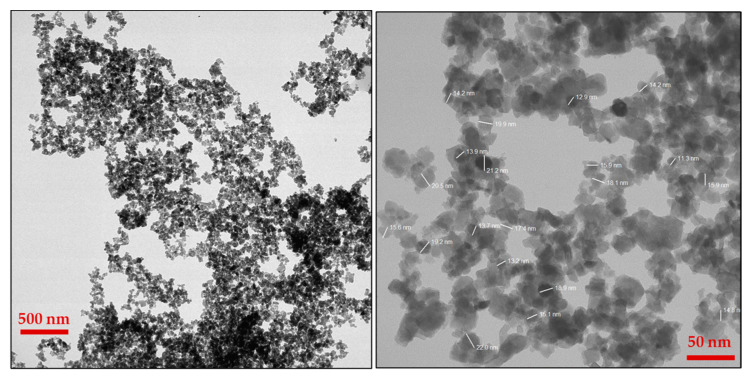
Images of TEM at different magnifications.

The synthesized WO_3_ NPs were also investigated through elemental and morphological analysis using microscopic techniques. The SEM, elemental mapping, EDX, and TEM results are shown in Figure 2, Figure 3, Figure 4 and Figure 5. The SEM image (Figure 2) represents the particle in a semi-speckled manner and as being rough. The EDS elemental mapping shows a uniform distribution of C, N, W, and O particles in the WO_3_ crystal, as shown in Figure 3a–d. The EDS spectrum of WO_3_ (Figure 4) reveals the peaks of 33.3% of tungsten (W), 57.5% of oxygen (O), 7.9% of carbon (C), and 1.3% of nitrogen (N). The observed peaks corresponding to W and O were attributed to the WO_3_ metal oxide, whereas signals C and N originated from the phytochemicals present in the PE. 

The morphology of WO_3_ particles was investigated using TEM, as shown in Figure 5. The TEM images revealed the presence of slight agglomeration; however, the agglomeration was minimal and did not significantly affect the dispersion of particles. The sizes of the particles were determined from TEM analysis, which were in the range of 10–25 nm. A uniform distribution of WO_3_ particles was further confirmed through SEM, elemental mapping, and TEM imaging. The SEAD analysis attached to the TEM instrument showed corresponding planes for WO_3_ (Inset Figure 1). The SEAD results were consistent with the XRD analysis, confirming the formation and crystallinity of WO_3_.

#### 3.1.3. Functional Group Analysis

**Figure 6:**
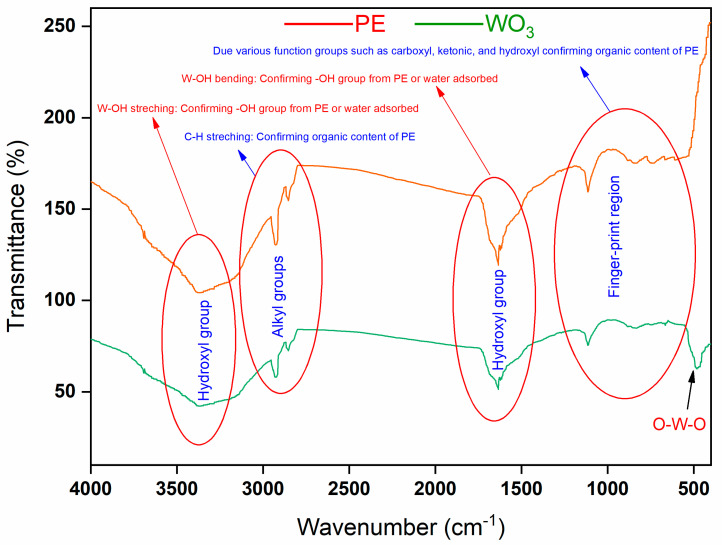
FTIR spectrum of PE (red line) and WO_3_ NPs (green line).

As the photocatalyst was synthesized via a green route using the PE, it is reasonable to assume that phytochemicals present in the PE contributed to the surface functionalization of the resulting NPs. The functionalization was investigated and confirmed through FTIR analysis. The FTIR spectra were recorded between 400 and 4000 cm^−1^ to identify the characteristic functional group (Figure 6). The FTIR spectrum of the PE (blue line) displays the prominent functional group peaks of the hydroxyl, carbonyl, and alkyl groups, which are attributed to phytochemicals present in the PE. In the FTIR spectrum of NPs (black line), along with these groups, there is an additional distinct peak at 491 cm^−1^, which is characteristic of the tungsten–oxygen bond. This analysis confirms that there are synthesized W-oxygen NPs, with the functionalized surface being derived from phytochemicals in the PE.

#### 3.1.4. Band Gap Calculation

**Figure 7:**
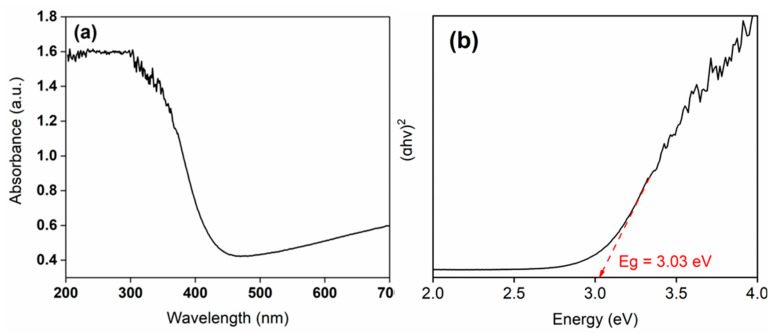
Plot of (**a**) UV–Vis absorption spectra of WO_3_ and (**b**) Tauc’s results for the calculation of energy band gap (Eg) of WO_3_.

The optical properties of the synthesized WO_3_ photocatalyst were investigated through UV–Vis spectroscopy, and its band gap energy was subsequently determined (Figure 7a,b). As shown in Figure 7a, WO_3_ exhibits a distinct absorption edge around 400 nm, which is consistent with the UV–Vis spectra of WO_3_ reported in the previous literature [60,61,62]. 

Typically, the optical band gap of WO_3_ has been reported in the range of 3.0 to 3.4 eV. For instance, Ahmad et al. [63] reported an optical band gap of 3.36 eV for a sputtered single-layer WO_3_. In the present study, the calculated band gap of the synthesized WO_3_ catalyst was ~3.0 eV, which is consistent with the previously reported value of the WO_3_-based photocatalyst [64]. 

The relatively lower band gap observed in the present study may be attributed to the biochemical synthesis route employed for photocatalyst preparation. Similar trends have been reported in previous studies, where biologically derived photocatalysts exhibited band gap values conducive to visible light absorption. The obtained band gap value confirms the suitability of the synthesized WO_3_ for photocatalytic applications. Consequently, methylene blue (MB) dye was selected as a model contaminant to evaluate the photocatalytic degradation efficiency of the prepared material.

### 3.2. Results of Photocatalytic Degradation

**Figure 8:**
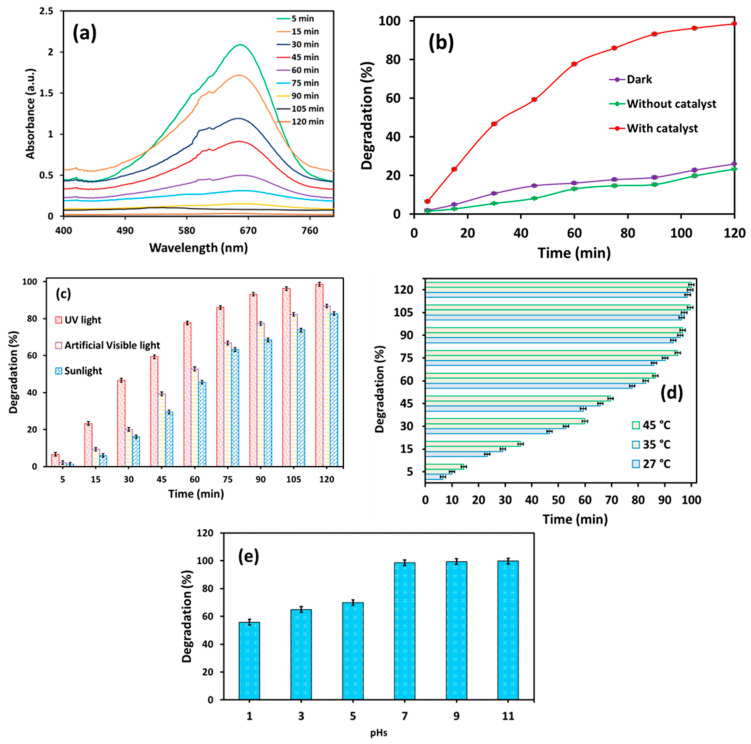
Results of time-dependent study of photocatalytic degradation of MB: (**a**) UV–Vis spectra of MB degradation under UV light irradiation; (**b**) estimation of MB degradation (%) under the condition of darkness (with WO_3_), with and without WO_3_ (under UV light irradiation); (**c**) effect of temperature on MB degradation (under UV light irradiation using WO_3_); (**d**) comparative analysis of MB degradation under UV light, visible light, and sunlight irradiation; and (**e**) effect of pH on MB degradation (under UV light irradiation using WO_3_).

The photocatalytic degradation of MB was monitored at 15-min intervals over a total duration of 120 min under UV light irradiation using a synthesized WO_3_ photocatalyst (Figure 8a). To evaluate the role of different irradiation sources and conditions, comparative experiments were conducted under UV light without WO_3_, with WO_3_ under dark artificial visible light, and under natural sunlight conditions (Figure 8b,c).

As represented in Figure 8b, WO_3_ exhibited the highest degradation (~99% degradation efficiency in 120 min) under UV light irradiation. This performance significantly surpassed that observed under visible light (~87%) and sunlight (~83%) irradiation under otherwise identical conditions (Figure 8c). In contrast, the MB degradation efficiency was nominal under dark conditions (Figure 8b). Similarly, the MB degradation (Figure 8b) in the absence of WO_3_ under UV light irradiation was negligible, further confirming the critical role of the WO_3_ catalyst in the photodegradation process. These MB degradation results confirm the effective photodegradation activity of WO_3_ under UV light. However, it is important to consider that visible light and sunlight are more sustainable and cost-effective sources of light than UV light. In addition to photocatalytic efficiency, the thermodynamic and kinetic aspects were also investigated to better understand the underlying degradation mechanisms.

#### 3.2.1. Effect of Temperature

The influence of temperature on MB degradation was investigated at three temperatures: 27 °C, 35 °C, and 45 °C. The corresponding results are presented in Figure 8d. As evident from the figure, the MB photocatalytic degradation increases with rising temperature under UV light irradiation. These trends align with the previous report [65], which suggests that higher temperature promotes the formation of bubbles, thereby increasing the generation of free radicals and furthermore enhancing MB degradation. Additionally, the increase in temperature reduces electron–hole recombination and enhances the oxidation of MB rates.

#### 3.2.2. Effect of pH

The effect of pH on photocatalytic degradation is a critical factor as natural water systems often experience fluctuations in pH, which can significantly influence photocatalytic activity. To evaluate this effect, the photodegradation activity was examined between 2 and 10 pHs under the same experimental conditions, and it is presented in Figure 8e. As shown in Figure 8e, the photodegradation activity increases with the increase in pH. The enhanced activity under alkaline conditions can be attributed to the increase in the production of hydroxide free radicals increases at a higher pH, and these enhanced •OH free radicals are capable of photocatalytic degradation at a higher rate [66]. These findings are further supported by the free radical scavenging experiment, which confirms the role of hydroxyl radicals in enhancing degradation at a higher pH.

#### 3.2.3. Results of Kinetics

**Figure 9:**
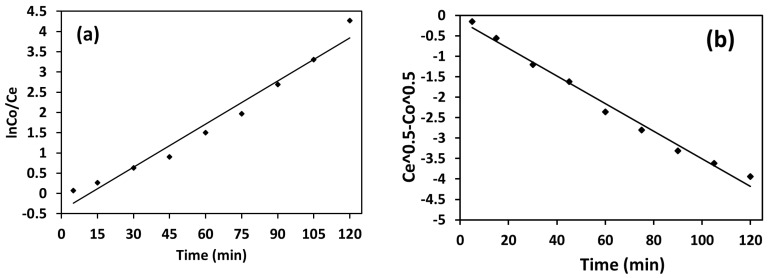
Results of kinetic study (**a**) pseudo-first-order and (**b**) diffusion model for photocatalytic degradation of MB using WO_3_.

Figure 9a,b represent the kinetic graphs obtained by kinetic models. As illustrated in Figure 9a, the degradation data exhibited a linear fit with the pseudo-first-order kinetic model, yielding a rate constant k_1_ value that is +0.0355 and *R^2^* = 0.969, indicating the reaction-first first-order kinetics. In contrast, the diffusion kinetic model produced a negative rate constant k value (*k_2_* = −0.0168), suggesting poor fit and eliminating the diffusion model (Figure 9b). The fitting of first-order kinetics proves that a chemical change occurs in the current photodegradation of MB dye.

#### 3.2.4. Results of Interfering Ions

**Figure 10:**
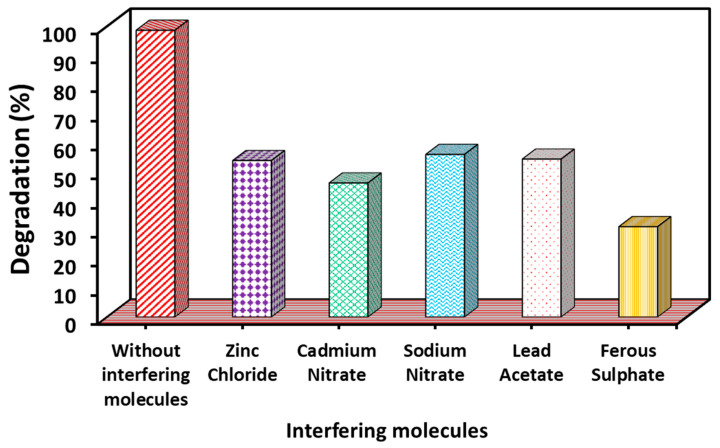
Effect of interfering molecules on MB degradation under UV–Vis light irradiation (experimental conditions: MB concentration = 20 mg/L; WO_3_ dose = 2 g/L; pH = 7; temperature = 27 °C; time: 120 min).

Interfering ions have a significant impact on dye degradation, which varies to a large extent. This is mainly because interfering ions (cations and anions) can act as scavengers for reactive free radicals, electrons, and holes that participate in the degradation process. Scavenging the reactive species ultimately prevents the degradation of dyes [66,67]. In addition, these interfering ions can also act as competitive ions that compete with the dye for adsorption on the photocatalyst surface. This competition leads to a decrease in the degradation activity. Therefore, for the present study, the MB dye degradation rate was also investigated under the same conditions in the presence of several interfering molecules (with the concentration of each interfering molecule being 10 mg/L) such as ZnCl_2_, Cd(NO_3_)_2_, NaNO_2_, Pd(CH_3_COO)_2_, and FeSO_4_. It was found that all these ions significantly reduced the rate of MB degradation (Figure 10).

#### 3.2.5. Results of Regeneration Experiment

**Figure 11:**
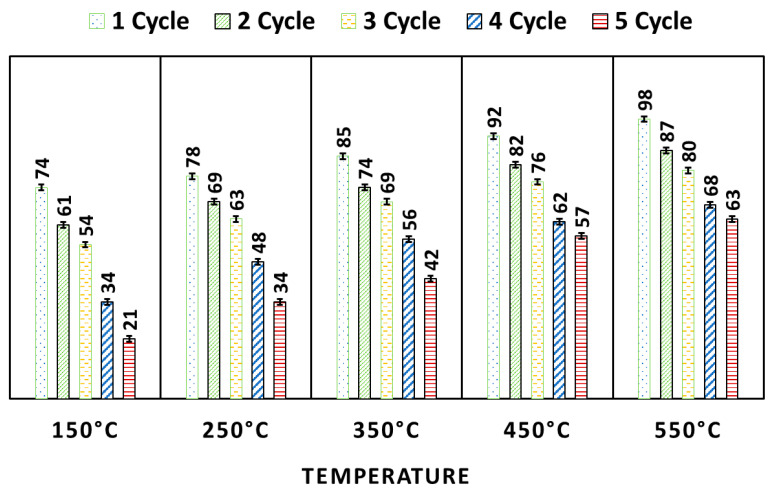
Results of regeneration and reusability test for WO_3_.

On using regenerated WO_3_, it is found that as the temperature of heating increases (150 to 550 °C), its degradation efficiency also increases (Figure 11). This is because the high temperature is very efficient for the pyrolysis of MB, adsorbs on the surface of WO_3_, and increases the free surface sites, and it is ultimately efficient for the higher degradation efficiency of WO_3_. All five pyrolyzed photocatalysts were used for several cycles. The photocatalysts heated at all five temperatures showed good reusability. The pyrolyzed WO_3_ (at 550 °C) showed 63% MB degradation after the fifth cycle. This result suggested the high stability of the WO_3_ NP.

#### 3.2.6. Result of Free Radical Scavenging

**Figure 12:**
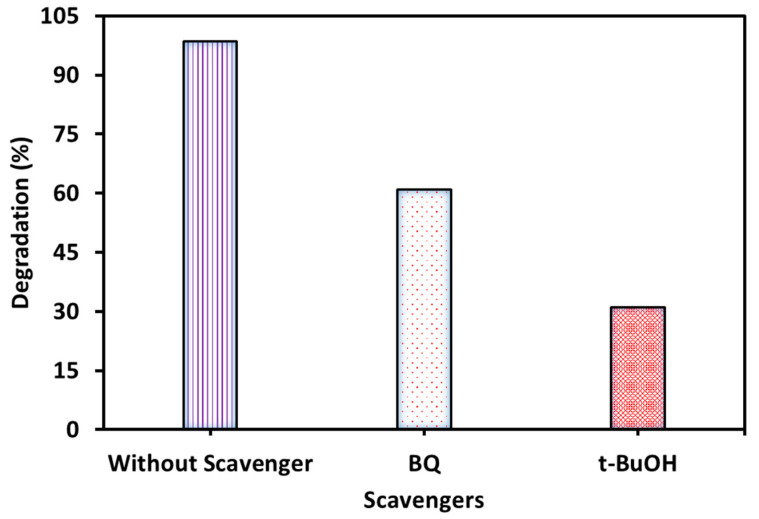
MB degradation occurs in the presence of scavengers (experimental conditions: MB concentration = 20 mg/L, WO3 dose = 2 g/L, pH = 7, temperature = 27 °C, time = 120 min).

The free radical scavenging results are shown in Figure 12. It can be observed from the results of the free radical scavenging experiment that a higher photodegradation rate was found in the presence of BQ than in the presence of t-BuOH. This result confirms that the •OH free radical was more active for MB photocatalytic degradation than •O_2_−.

#### 3.2.7. Mechanism

**Figure 13:**
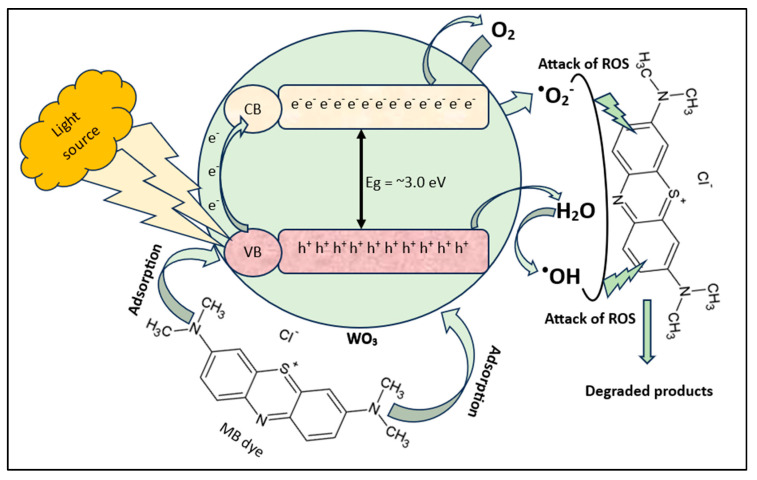
Proposed mechanism for the photodegradation of MB using WO_3_.

The results of the scavenging experiment show that superoxide free radicals are partial while •OH ones are effectively responsible for the degradation of dye. Therefore, in the present study, the mechanism of MB degradation can be explained by the free radical generation produced during the photodegradation process. WO_3_ is a catalyst having a valence band (VB) and conduction band (CB) between which electrons are transferred. Since WO_3_ is synthesized by a biological process using the PE, WO_3_ therefore contains various functional groups. Initially, MB molecules are adsorbed on the WO_3_ surface containing functional groups. When light falls on the surface of WO_3_, it excites electrons from the VB. The excited electrons move to the CB and leave positive holes (h^+^) in the VB. The conducted electrons (present in the CB), reaching the surface of WO_3_, interact with O_2_ molecules and generate •O_2_−. On the other hand, positive holes in the VB have very high oxidizability, and they react with water molecules and produce •OH. The generated free radicals (called reactive oxygen species) collide with the MB molecules and break them down into various simpler components, causing MB to decompose into non-toxic substances. The present MB degradation pathway is shown in Figure 13, which is confirmed by many previous studies [65,68,69,70]. The proposed reaction steps based on the literature are given below (Equations (5)–(11)).

WO_3_ + hν → WO_3_ (e + h) (5)

WO_3_ (h) + H_2_O → WO_3_ + H^+^ + •OH (6)

WO_3_ (e) + O_2_ → WO_3_ + •O_2_^−^ (7)

•O_2_^−^ + H_2_O → •OOH + OH^−^ (8)

2 •OOH → O_2_ + H_2_O_2_ (9)

H_2_O_2_ + •O_2_^−^ → OH^−^ + OH + O_2_ (10)

•OH + MB → Degraded product(11)

## 4. Conclusions

In this study, the WO_3_ photocatalyst was successfully synthesized via a one-step bio-precipitation method using the PE. The resulting NPs exhibited a band gap of about 3.0 eV and a particle size in the range of 10 to 25 nm. The UV–Vis spectra demonstrated that the WO_3_ catalyst is capable of absorbing both the UV and visible range, making it suitable for photocatalytic applications under a broad range of irradiation conditions. Photocatalytic degradation experiments using methylene blue dye showed that WO_3_ achieved up to 99% under UV light within 120 min. Although slightly lower, significant degradation efficiencies were also observed under visible light (~87%) and sunlight (~83%). Free radical scavenging experiments confirmed that hydroxyl free radicals were dominantly responsible for the methylene blue photodegradation, while superoxide free radicals were to a lesser extent. The presence of interfering ions was found to inhibit degradation. Thermal treatment was carried out to investigate the regeneration and reusability efficiency of WO_3_. It was found that the pyrolyzed WO_3_ at 550 °C showed remarkable stability, maintaining 63% MB degradation after five cycles. Overall, the bio-derived WO_3_ photocatalysts demonstrate high thermal stability, the fast and complete degradability of methylene blue dye, and reusability, making them a promising material for sustainable photocatalytic applications.

## Data Availability

The data that support the findings of this study are available from the corresponding authors, upon reasonable request.
